# Increased Risk of Decompression Sickness When Diving With a Right-to-Left Shunt: Results of a Prospective Single-Blinded Observational Study (The “Carotid Doppler” Study)

**DOI:** 10.3389/fphys.2021.763408

**Published:** 2021-10-29

**Authors:** Peter Germonpré, Pierre Lafère, William Portier, Faye-Lisa Germonpré, Alessandro Marroni, Costantino Balestra

**Affiliations:** ^1^Centre for Hyperbaric Oxygen Therapy, Military Hospital, Brussels, Belgium; ^2^Divers Alert Network (DAN) Europe Research Division, Roseto, Italy; ^3^Environmental & Occupational, Ageing (Integrative) Physiology Lab, Haute Ecole Bruxelles-Brabant (HE2B), Auderghem, Belgium; ^4^Physical Activity Teaching Unit, Motor Sciences Department, Université Libre de Bruxelles (ULB), Brussels, Belgium

**Keywords:** decompression sickness, prospective study, relative risk, right-to-left shunt (RLS), patent foramen ovale (PFO), adverse effects, SCUBA dive, diving

## Abstract

**Introduction:** Divers with a patent Foramen Ovale (PFO) have an increased risk for decompression sickness (DCS) when diving with compressed breathing gas. The relative risk increase, however, is difficult to establish as the PFO status of divers is usually only determined after a DCS occurrence.

**Methods:** This prospective, single-blinded, observational study was designed to collect DCS data from volunteer divers after screening for right-to-left shunt (RLS) using a Carotid Doppler test. Divers were blinded to the result of the test, but all received a standardized briefing on current scientific knowledge of diving physiology and “low-bubble” diving techniques; they were then allowed to dive without restrictions. After a mean interval of 8 years, a questionnaire was sent collecting data on their dives and cases of DCS (if any occurred).

**Results:** Data was collected on 148 divers totaling 66,859 dives. There was no significant difference in diving data between divers with or without RLS. Divers with RLS had a 3.02 times higher incidence of (confirmed) DCS than divers without RLS (*p* = 0.04). When all cases of (confirmed or possible DCS) were considered, the Relative Risk was 1.42 (*p* = 0.46). DCS occurred mainly in divers who did not dive according to “low-bubble” diving techniques, in both groups.

**Conclusion:** This prospective study confirms that DCS is more frequent in divers with RLS (such as a PFO), with a Relative Risk of 1.42 (all DCS) to 3.02 (confirmed DCS). It appears this risk is linked to diving behavior, more specifically diving to the limits of the adopted decompression procedures.

## Introduction

Since the early 1990’s, patency of the Foramen Ovale of the heart (Patent Foramen Ovale, PFO) has been identified as a risk factor for decompression sickness (DCS) in self-contained underwater (SCUBA) diving (Moon et al., 1989; Wilmshurst et al., 1989). Numerous case reports have illustrated so-called “undeserved DCS,” DCS occurring after dives within the accepted decompression limits and without violation of accepted decompression procedures, to be associated with PFO (Germonpre et al., 1998; Sykes and Clark, 2013). Several attempts have been made to quantify the increased risk of diving for divers with a PFO based on retrospective diving accident data (Bove, 1998; Torti et al., 2004). In these “risk-comparison” papers, a 2.9 (Bove, 1998) to 5.7 times higher risk (Torti et al., 2004) for DCS has been reported, although the latter figure has been criticized based on the subjective definition criteria used for DCS (Germonpre and Balestra, 2004).

This retrospective approach is only a rough approximation of the actual increase of the risk, for several reasons. First, because the total number of dives performed is not known, the denominator of the risk equation is missing. Secondly, the type of diving influences the risk for DCS, with certain diving behavior – such as deep decompression diving, technical diving – yielding a significantly higher risk than no-decompression recreational diving (Germonpre, 2006). Third, DCS is characterized by a highly variable spectrum of symptoms, making the diagnosis often difficult; both over-reporting and underreporting are frequently observed (Steffensmeier et al., 2017; Hubbard et al., 2018). Lastly, the diagnostic accuracy of PFO detection is highly dependent on the technology and technique used for PFO detection (Gin et al., 1993; Wilmshurst et al., 2003; Attaran et al., 2006; Johansson et al., 2010). Unless all these factors can be accounted for, estimates of increased DCS risk remain highly speculative (Vann et al., 2008; Wilmshurst, 2019).

With the increased availability and documented safety of percutaneous closure devices for PFO, interventional therapy appeals to more and more divers as a simple and safe cure for “the PFO problem.” Indeed, studies have shown that closing the PFO reduces the risk of paradoxical embolization of decompression bubbles (Honěk et al., 2014a) and also of decompression sickness (Billinger et al., 2011; Honěk et al., 2020). On the other hand, adhering to more “conservative” diving profiles has also been shown to reduce the risk, in a similar degree (Klingmann et al., 2012; Honěk et al., 2014b). Percutaneous PFO closure carries a small but non-negligible risk of procedural complications, as well as significant costs to be carried by the patient or by public money (Social Security); adopting safer diving profiles mainly restricts the recreational diver psychologically (the feeling of “being limited”). Generally, PFO closure in the context of diving is not regarded as a medical necessity, except perhaps for professional and military divers (Pristipino et al., 2021).

In order to help divers and diving medicine physicians decide which approach is the best in a particular case, determining the relative risk in a more precise way, is important. We aimed to perform a prospective, single-blinded study to determine the relative risk (RR) of diving with a right-to-left shunt (RLS).

## Materials and Methods

### General Methodology

The feasibility of such a study needed to be ascertained first. A power analysis was made based on available retrospective data. We had to develop a screening test that we could use on a large number of divers without any problems. We had to address the fact that if we test divers and we tell them whether they have a right-to-left shunt or not, they might change their diving behavior and so “falsify” the study by diving much safer then they normally would. Inversely, divers who were told they do not have a shunt might feel less “vulnerable” and less restricted to perform more “risky” dives (in an extreme scenario, we could end up seeing divers without RLS having more DCS than divers with RLS). Ethical committee approval was needed, not only for the screening test but also for the fact that divers would be blinded to the result of the test. We had to take into account the probability of a large number of drop-outs (“lost to follow-up”) as the study would take years to complete. Finally, we would have to make sure that data were collected with a maximum of accuracy, both regarding exposure (number and types of dives performed) and outcome (absence or presence of DCS).

### Sample Size Calculations and Statistics

We assumed the Odds Ratio for DCS when having RLS to be 4, which is higher than reported by Bove (1998) but lower than was later reported in the Swiss study (Torti et al., 2004). We assumed the prevalence of PFO in the general divers’ population to be 25 percent, as reported in the seminal Mayo Clinic autopsy study (Hagen et al., 1984). Even though a PFO seems to be more prevalent in the young and less prevalent in older people, having a single rounded number is convenient for these sample size calculations.

We assumed a general risk of DCS for divers without a PFO to be 1 in 10,000 dives. For our “best case scenario,” with a 95% power of the study and no dropouts at the end of the study, we needed a total of 200,792 dives or 803 divers, each performing an average of 50 dives/year over 5 years. In a “worst case scenario” with a 50 percent dropout and a power of only 80%, we would need 235,712 dives (or about 940 divers for a period of five years).

Results are analyzed with descriptive statistics, Student *t*-test after checking for normality, or proportion analysis where appropriate.

### Development of the Screening Test

In order to be an acceptable screening test for RLS, it needs to be minimally invasive, low tech, low cost, and have a good sensitivity/specificity ratio. In 1999, we developed and described the Carotid Artery Doppler test (Germonpré et al., 1999; Wendling et al., 2001). In short, a large antecubital intravenous catheter was placed in the right elbow fold, and connected to a short tubing with two three-way valves attached. An intravenous injection of 9.5 mL of normal saline solution with 0.5 mL of air, agitated by pushing to and fro between two 10 mL syringes, was performed at the end of a respiratory “straining” maneuver. This is similar to the procedure used during a classic contrast echocardiography for detection of PFO. The “straining” maneuver (often erroneously called a Valsalva maneuver) consisted of a voluntary intrathoracic pressure increase (blocking the respiration and “bearing down”) for ten seconds, followed by an abrupt release of pressure by exhaling. The agitated saline solution was rapidly injected just before release of the straining. Using an 8 MHz vascular Doppler device placed over the left carotid artery, a “gurgling” bubble sound could be heard over the regular arterial Doppler sounds, in case of a RLS. The test subject was blinded to those sounds using headphones with loud music.

This test was validated on a group of 33 patients, in a single blinded comparison with contrast transesophageal echocardiography, and yielded a very good sensitivity (100%) and specificity (88%) with only a few “false positives” (as doubtful signals were to be classified positive) Wendling et al., 2001). A French group (Blatteau, 1999) replicated our validation test on 200 patients with transesophageal contrast echo, and found similar good figures for sensitivity and specificity (sensitivity 89%, specificity 97%).

**FIGURE 1:**
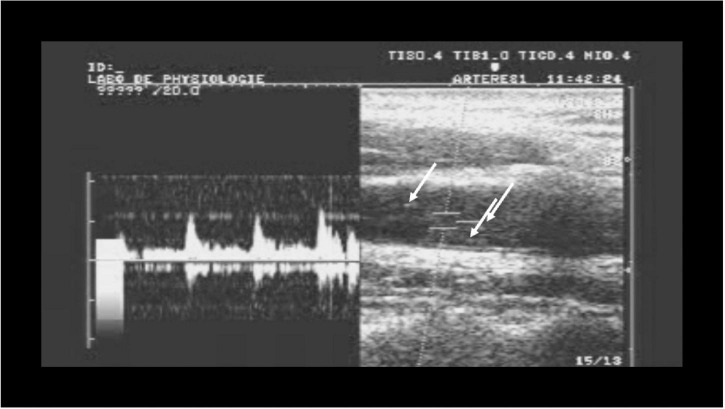
Simultaneous Pulsed Wave Doppler-mode and B-mode ultrasound over the carotid artery, showing bubbles (arrows) passing through the imaging field producing the “gurgling” sound heard over the carotid artery with Doppler.

During the development of the test, we verified that the sounds that are heard are actual bubbles passing into the carotid artery. Using 2-D duplex scanning, bubbles could be seen passing the beam of the Doppler probe and producing the distinctive sound (Figure 1).

### Recruitment of Investigators and Subjects

A dedicated page on the DAN Europe website was set up to recruit investigators, who were all diving medicine physicians. We developed a study package consisting of a PowerPoint presentation, printed forms, report files, and injection materials, if needed. We organized “Carotid Artery Doppler” training workshops, because, even if the test was relatively easy to perform, training was needed to make sure there were no “false negatives.” All the materials were developed in Dutch, French, English, German, and Italian, those being the primary target areas in Europe (in Europe, over 25 different languages are spoken, so we would only develop specific language materials if a sufficiently high yield was expected). During the workshop, every investigator received a hands-on training on about 10 divers to ascertain they could reliably report the results of the test. Furthermore, round the clock telephone and e-mail support was provided.

We used the DAN Europe website and personal contacts to recruit divers and organized “research sessions” for about 10 to 12 divers at a time. First, these divers were given a 1.5 h long lecture, illustrated by the Powerpoint presentation, on diving decompression risks. During this lecture, information was given on how the risk for DCS is dependent, not only on gas load but also on many other, often unknown factors; about PFO and how it would increase the risk for DCS; on the importance of conservative, “low-bubble” diving to reduce the risk of DCS. Then they were informed again that, while they would be tested for RLS, they would not be told the result of the test; and we would simply encourage them to “dive safely” (Wendling et al., 2001).

### Testing

**FIGURE 2:**
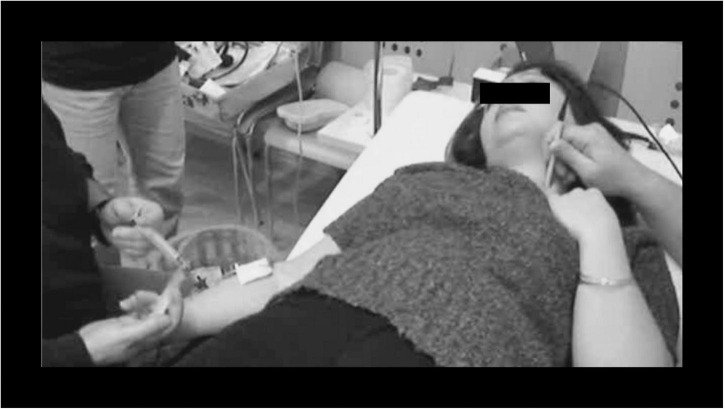
Carotid Doppler test. Right arm: injection of contrast medium (10 mL of agitated saline solution); Left carotid artery: 8 MHz Doppler probe.

Ethical Committee approval was obtained (Bioethics Committee of the Belgian Defense Force Medical Staff, 2003), and divers signed an informed consent form prior to the testing. Each testing was performed individually, subjects going through the informed consent process one at a time. During the test, subjects wore headphones with loud music so they couldn’t hear the Carotid Doppler signals. First, we did a few “simulated injections,” to practice the straining maneuver. Then up to three saline injections with a properly performed straining maneuver were performed (Figure 2).

After the test, divers were again reminded of the importance of “safe diving” and were provided with a “Carotid Doppler Study Participant” card, with a telephone number and an e-mail address. They were asked to inform the investigators in case of a diving accident (but not as an emergency phone number) and also if they moved or changed telephone or e-mail address. They were also informed that approximately 5–6 years after the test, they would be contacted again to provide information on their dives and diving incidents or accidents.

## Results

The study had a recruitment period of about eight years (2001–2009). During that period, 11 investigators were recruited and trained, but only 6 have provided final data. Four provided data on less than 10 divers after their initial training. Three of the investigators had decided not to blind the divers to the result, thus deviating from the study protocol. These “non-blinded” data were analyzed separately.

Four hundred and forty-five divers were recruited, of which 55% were effectively blinded to the result. The mean age of the test subjects was 38.3 years, and 32% were female. In 18.9% of subjects, the Carotid Doppler test was positive, indicating a RLS. This was the initial data collection.

Each participating diver was contacted again after a period of between 7 and 10 years, and was sent a “final questionnaire” enquiring on their diving experience since the test (number and types of dives), the DCS or diving incidents they might have had, and a number of other questions to make the questionnaires both detailed but not too cumbersome to fill in. The questionnaire can be found in the Supplementary Materials.

As expected, a large number of divers could not be located anymore, even though telephone numbers, postal address, e-mail and other data had been recorded. Only 33.3 percent of participants returned the questionnaire (148 divers). Efforts to retrieve more questionnaires were continued through the end of 2019.

The majority of the return data were received from “blinded” divers. Of the “non-blinded” divers only 14.5% responded, as opposed to 46.12% of the “blinded.” There were some divers who stopped diving after the test, and that may have been or may not have been because the test was positive. Two divers had their PFO closed after detecting it with the Carotid Doppler test.

The total number of dives collected was 66,859. Demographics (see Table 1) showed no differences between RLS-negative and RLS-positive divers. There were no significant differences in sex distribution, age, height and weight, smoking habits, or study duration (7.94 vs. 7.96 years). The diving experience before and the number of dives after the tests was not significantly different, although RLS positive divers tended to have slightly fewer dives before the test (441 vs. 524 dives, NS).

**TABLE 1:** Biometry and dive experience.

	**RLS Positive**	**RLS Negative**	**p-value**
**n**	**28**	**120**	
Males (%)	71.4	80.0	ns
Age (mean ± SD)	35.0 ± 8.7	39.1 ± 11.3	ns
Smokers (%)	10.7	14.2	ns
Duration of study (years)	8.0	7.9	ns
Height (cm, mean ± SD)	171.6 ± 22.0	178.5 ± 12.5	ns
Weight (kg, mean ± SD)	76.7 ± 14.0	82.7 ± 24.7	ns
Dive experience before test (number of dives) (mean ± SD)	441.0 ± 751.2	524.7 ± 843.56	ns
Average number of Dives/year before test (mean ± SD)	59.1 ± 82.0	55.4 ± 36.1	ns
Total number of dives since test	15.494	51.365	ns
Total hours diving since test	11.827	41.043	ns
Dives/subject since test (mean ± SD)	553,4 ± 712.2	428.0 ± 467.6	ns

Most divers used dive computers for decompression management, most of them used the popular dive computer brands (Uwatec, Suunto). Some divers still used tables, some divers used “technical” dive computers, but the majority could be considered recreational divers (Table 2). This reflects the recreational diving population characteristics in the first decade of this century. In the final questionnaire we also inquired what kind of dives they had done during the study period. We arbitrarily divided the dives into “decompression air dives,” “no-decompression air dives,” “decompression nitrox dives,” “no-decompression nitrox dives,” “technical dives” and “nitrox on air tables/computer” dives, providing a detailed description of each type of diving activity. We also tried to make a second categorization of the dives, between “recreational dives,” “sports dives” (deep square dives, such as on wrecks or deep reefs), “deep dives” (over 40 meters), and “low risk dives.” Again, there is no significant difference between the two groups in this respect.

**TABLE 2:** Type of diving performed during study period (for description of types of dives, see article text).

	**RLS Positive**	**RLS Negative**	**p-value**
Main dive computer used: UWATEC (%)	53.6	53.3	ns
Main dive computer used: SUUNTO (%)	39.3	28.3	ns
Main dive computer used: Other (%)	0.0	10.0	ns
Dive table use (%)	3.6	13.3	ns
Total number of dives since test	15.494	51.365	ns
Decompression Air Dives (%)	20.10	23.67	ns
No Decomp Air Dives (%)	66.05	60.02	ns
Decompression Nitrox Dives (%)	3.02	3.47	ns
No Decomp Nitrox Dives (%)	5.97	7.62	ns
Nitrox Dives Using Air Tables (%)	1.56	2.95	ns
Other Breathing Gas/Technical diving (%)	5.12	5.75	ns
“Recreational” Dives	27.53	30.98	ns
“Sports” Dives	40.21	42.70	ns
“Deep” Dives	22.50	19.68	ns
“Low Risk” Dives	12.54	14.46	ns

A total of 8.3% of RLS negative divers and 28.6% of RLS positive divers had experienced a confirmed episode of DCS during the study period. The incidence of DCS per 10,000 dives was 1.95 and 5.16, respectively, yielding a Relative Risk of 2.65 (CI 1.05 to 6.72, *p* = 0.039). Some divers reported symptoms, not having been treated as DCS and a detailed description of these symptoms failed to positively identify those as DCS. These were classified as “possible DCS.” If also those cases of “possible DCS” are taken into account, the incidence in RLS negative divers is 15.8% and in RLS positive divers 32.1%. The incidence per 10,000 dives is a little more than 1.5 times higher for RLS positive divers (5.81/10,000 dives vs. 3.70/10,000 dives), giving a RR of 1.57 (CI 0.71 to 3.47, p = 0.26) (Table 3A).

**TABLE 3A:** Decompression sickness events (all divers).

	**RLS Positive**	**RLS Negative**	**p-value**
**n**	**28**	**120**	
Confirmed DCS (n)	8	10	
DCS (%)	28.6	8.3	
DCS incidence per 10,000 dives	5.16	1.95	0.039
Possible DCS (n)	1	9	
Total (confirmed + possible DCS) (%)	32.1	15.8	
Total incidence per 10,000 dives	5.81	3.70	0.26
HBO treatment for DCS (n)	4	5	
Skin DCS (cutis marmorata) (n)	4	9	
Vestibular/cochlear DCS (n)	6	3	
Spinal cord DCS (n)	1	5	
			

As stated, of the 148 divers who returned the Final Questionnaire, only a minority were “non-blinded” (21 of 148, 18%). Analysis of only the “blinded” divers, unsurprisingly, does not change much to the previous analysis. For confirmed DCS, the incidence was 7.6% vs. 27.3%, yielding an incidence of 1.8 and 5.46 DCS per 10,000 dives, respectively (for a Relative Risk of 3.02; CI 1.0502 to 8.7198, *p* = 0.040). For “all DCS, confirmed and possible,” the incidence was 16.2% vs. 27.3%, which amounts to 3.83 and 5.46 DCS per 10,000 dives (Relative Risk 1.42; CI 0.5616 to 3.6110, *p* = 0.46) (Table 3B).

**TABLE 3B:** Decompression sickness events (“blinded” divers only).

	**RLS Positive**	**RLS Negative**	**p-value**
**n**	**22**	**105**	
Confirmed DCS (n)	6	8	
DCS (%)	27.3	7.6	
DCS incidence per 10,000 dives	5.46	1.80	0.040
Possible DCS (n)	0	9	
Total (confirmed + possible DCS) (%)	27.3	16.2	
Total incidence per 10,000 dives	5.46	3.83	0.46
HBO treatment for DCS (n)	2	3	
Skin DCS (cutis marmorata) (n)	3	8	
Vestibular/cochlear DCS (n)	3	2	
Spinal cord DCS (n)	1	5	

Regarding the types of symptoms of DCS, it is interesting to note that although cutaneous symptoms [cutis marmorata or livedo racemosa (Hartig et al., 2020)] and vestibular or cochlear DCS are most commonly associated with the presence of arterialized gas bubbles (PFO) (Germonpre et al., 1998; Wilmshurst et al., 2001; Cantais et al., 2003), it appears to be a symptom of (possible) DCS in RLS negative divers as well. Spinal cord decompression sickness also occurred (once) in shunt-positive divers.

Scrutiny of the DCS cases (for a more detailed description see the Supplementary Materials) revealed that all of the RLS positive divers that were treated with recompression had performed very deep dives, to a depth of 58, 54, 36, and 65 m, respectively (the 36 msw dive was a closed-circuit rebreather – CCR – dive with 50 min of bottom time).

The dives of those cases that were not treated with recompression were, likewise, more provocative than can be expected from recreational diving. One diver had severe vertigo and nausea after a 99 msw dive on air. Other DCS cases in the RLS positive group were repetitive decompression dives, a square dive to 62 msw for 68 min on CCR, square decompression cold water dives.

In the shunt-negative group, 4 treated DCS occurred after deep trimix technical dives or square decompression cold water dives (2 on CCR, 2 “open circuit”). Thirteen dives resulting in untreated post-dive symptoms were on average 40 msw depth, which is at the limits of “recreational diving”; six of them were repetitive or decompression dives. Only two of those divers applied oxygen first aid, indicating that divers’ denial is still very much present despite proper “education” (Lafere et al., 2017).

## Discussion

This is the first, and to our knowledge to date the only prospective evaluation of the Relative Risk (RR) of DCS when diving with RLS. It suggests that the risk for (confirmed) DCS is 2.65 times higher in divers with a RLS (*p* = 0.039). If we take into account all reported symptoms possibly associated with DCS, the RR is 1.57, which statistically is non-significant (*p* = 0.26). These figures are in line with previous, retrospective reports. However, it must be noted that most decompression sickness cases in the RLS positive group occurred after dives that are beyond reasonably defined recreational diving safety limits.

### Strengths and Weaknesses

There are several strengths and weaknesses to our study.

First, the Carotid Doppler test detects right-to-left shunts without actual imaging of the inter-atrial septum. It is thus possible that some divers had in fact intrapulmonary shunts, not a PFO (Madden et al., 2015). However, in both validation studies (Blatteau, 1999; Germonpré et al., 1999) no pulmonary shunting was observed during transesophageal echocardiography, making the possibility of a significant “PFO-negative / Carotid Doppler-positive” case number, very low. Furthermore, it is of little importance for divers whether RLS occurs through a PFO or a pulmonary shunt, as the end result (arterialized VGE) is the same. Finally, it would be anyhow improper to perform a PFO closure procedure without first verifying the morphology of the interatrial septum by echocardiography.

Secondly, at the end of 2019, when the data collection was definitively closed as it did not yield any further data, only 33.26 percent of all the divers had responded. According to our initial sample size calculations, the study would still be largely under-powered: with the assumed DCS incidence of 1 in 10,000, the power is only 40%, However, the DCS incidence in our cohort was found to be much higher than the initially assumed incidence (2.53 per 10,000 dives). This reflects the incidence reported in other papers for “cold water sports diving” (Bove, 1998; Germonpre, 2006; Cialoni et al., 2017). If the sample size calculations are re-done using this actual incidence of DCS, then our study, as it is, has a 90% power. So even with the number of divers lower than expected, the study seems to have enough power to support our conclusions.

Thirdly, all confirmed and possible DCS cases were invited to perform a contrast transthoracic echocardiography. With proper saline contrast and straining maneuver technique, almost all PFO cases can be diagnosed on transthoracic echocardiography, obviating the need for transesophageal echocardiography (Wilmshurst, 2016). However, there are indications that patency of the Foramen Ovale may increase over time (Hagen et al., 1984; Germonpre et al., 2005). As the initial study period was already 7 years and by the end of the data collection, in some cases more than 15 years had passed since the initial Carotid Doppler test, a formal PFO detection would not necessarily be contributive: it would be expected that some divers who were initially “RLS negative” could now have become positive for PFO. In any case, only a minority declared to agree to this examination. This was thus not further pursued.

Fourth, 45% of our participants were, contrary to the protocol, not blinded to the results of the Carotid Doppler test. Analysis of these divers revealed that the response rate for non-blinded divers was significantly lower (14.5% vs. 48.57% in the blinded group), although the proportion of divers with DCS was similar in the non-blinded group than in the group that has been blinded (13.79% vs. 11.02% in the blinded group). At least two of the divers in the non-blinded group have decided to have their PFO closed subsequently to learning the Carotid Doppler test result. In one of the “non-blinded” groups, it was recorded that 6 out of 22 “RLS-positive” and 7 out of 49 “RLS-negative” divers had already suffered DCS prior to participating in the study. None of the divers of this group that responded to the final questionnaire (13 out of 71) reported DCS after having taken the Carotid Doppler test. This leads us to think that, indeed, if you inform a diver that a RLS has been detected, he/she might (subconsciously or consciously) adapt their diving behavior, be just a little bit more careful and may not have any or less decompression-induced vascular gas emboli. This has been published by Klingmann et al. (2012): by simply educating divers (with or without a PFO) about the risk for venous gas emboli and recommending “safe dive practices,” the risk of subsequent decompression sickness can be reduced to almost zero, even in those divers with large PFOs. It was independently confirmed by Honěk et al. (2014b) Recommendations for conservative diving (also called “low-risk diving,” “low-bubble diving”) have been published by several diving safety organizations (Smart et al., 2015; Divers Alert Network, 2016; SUHMS, 2016), and basically consist of reducing the inert gas saturation during the dive by diving less deep, less long, or by breathing a gas with higher oxygen content (“Nitrox”) while still using the dive computer as if air were breathed (“Nitrox on air profiles”). This increases the safety margin set by the dive computer (Souday et al., 2016) as these devices only take a limited number of parameters into account, and none of the physiological variations know to play a role in saturation and desaturation (Cialoni et al., 2017).

In this way, the mere fact of screening divers for PFO might induce a “safer” diving behavior, as an alternative to PFO closure. How long such behavior would persist, is not known. Also, those divers that have been informed that they have a RLS might be seeking PFO closure preventively (before any DCS has occurred) for instance because of their “risky” diving behavior, or even in anticipation of switching to more risky diving (technical diving) (Ljubkovic et al., 2010; Germonpre, 2015).

#### Strengths

This study is the first, and to our knowledge, the only prospective study to date to observe a large number of divers who were blinded to their “RLS-status” over a large number of years, collecting data about their diving behavior and DCS incidents. Although not perfect, this study sought to minimize selection bias (divers were not financially or otherwise compensated for their participation), reporting bias (all instances of – possible – DCS were collected and reported) and diagnostic bias (the Carotid Doppler test detects both RLS through a PFO and through other shunting pathways, with minimal “observer variability”).

It has been shown repeatedly that knowledge of a possible RLS influences the diving behavior (even after the shunt has been closed), therefore, a blinded study remains the only way to objectively ascertain the risk of DCS in diving attributable to a RLS (which is mostly through a PFO). Taking also into account the diving “habits” (deep, technical or purely recreational leisure diving) is likewise important, as the “base” DCS risk obviously will be different (Germonpre, 2006; Ljubkovic et al., 2010; Hubbard et al., 2018).

Secondly, this study provides valuable information to help divers take an informed decision whether or not to proceed to PFO closure. Indeed, more and more divers consult our and other diving medicine physicians’ practice asking for a PFO detection test or seeking advice after a PFO has been fortuitously detected (i.e., without a history of DCS). That divers are ready to consider a (minimally, but still) invasive procedure in order to increase their (feeling of) security while diving, is an important observation. At least two of the divers in our study population have done so, both from the “non-blinded” group. Extrapolating this to all RLS positive divers, potentially up to 25% of those could be requesting PFO closure, even without ever having had DCS. Whether this is a valid preventive measure depends both on the risk involved in the closure procedure (short-, medium-, and long-term) and the degree of protection from DCS the procedure affords.

Several studies have described a reduction of arterial gas emboli in divers who have had their PFO closed as opposed to divers with PFO (Honěk et al., 2014a), even in a prospective manner. In view of the presumed pathophysiological mechanism, it seems logical that closing the PFO would reduce the risk of DCS after provocative (VGE-producing) dives.

A 5-year follow-up study after closure of PFO in Swiss divers has been published and is often cited as evidence that PFO closure is effective (Billinger et al., 2011). At first glance, the DCS incidence reduction in this report looks impressive (0.5/10,000 vs. 35.8/10,000 dives), but the absolute numbers of DCS cases are very low (4 in the “PFO” group, 1 in the “Closure” group over a 5-year period). In fact, if only one diver in the “PFO” group would have decided that his/her symptoms were not DCS, or if one diver in the “Closure” group would have declared that some vague symptom he/she had experienced were probably DCS, there would have been no statistical difference between those two groups. Moreover, divers who did not have their PFO closed (the “PFO” group) performed almost 50% of their dives deeper than 40 msw, whereas only 30% of dives in the “Closure” group was deeper than 40 msw.

Divers with a PFO (closed or not) apparently did change their diving procedures somewhat after the diagnosis, by performing slightly less of the deep (>40 msw) dives and choosing more often Nitrox as a breathing gas (although it was not specified whether they used “Nitrox” or “air” decompression procedures).

In other studies it was also reported that even though the PFO was closed, a significant number of divers adopted a more conservative diving attitude (Koopsen et al., 2018; Anderson et al., 2019; Vanden Eede et al., 2019). A recent report (Anderson et al., 2019) described a better reduction of DCS incidence after PFO closure compared to “conservative diving,” but major methodological flaws have been pointed out by an accompanying editorial (Wilmshurst, 2019).

A recent study (Vanden Eede et al., 2019) describing the follow-up of 59 PFO closures over a 10-year period in a Belgian diving population has reported a significant number of recurring DCS (15%) despite the PFO closure. These occurred either because the PFO had not been completely closed, or because of extremely provocative dives. Also, the procedure had an incidence of complications and side effects comparable to other reports (Pristipino et al., 2019).

Honěk et al. (2021) very recently reported the results of a risk stratification strategy study whereby divers, screened for PFO were offered the choice between PFO closure or “conservative diving” in case of high-grade PFO. Although both groups had a decreased incidence of DCS during the follow-up period of 6.5 years (the “closure group” approaching the DCS incidence of those divers without PFO), the “high-grade PFO – conservative diving” group still had a higher incidence. The depths and dive profiles attained by all of these divers, however, are to be considered quite provocative for recreational diving (depths to more than 40 meters, on air or nitrox); moreover, the “conservative diving” recommendations apparently consisted only of recommending dive computer-“no-decompression diving” to a maximum of 40 m. It appears from the Supplementary Data of this paper that only 75.5% of the “high-grade PFO” divers actually followed these recommendations.

Closing a PFO can thus be considered effective in reducing the risk of DCS but should not be considered a “free ticket” to unrestricted provocative diving. Also, long-term complications of the closure procedure are reported sporadically, and their incidence in follow-up studies is significant (Abaci et al., 2013; Merkler et al., 2017). On the other hand, the effectiveness of recommending “conservative diving practice” is dependent on a continued diver education and could benefit from a “cultural change” in the diver community. For divers who cannot (e.g., professional divers) or do not wish to adopt (e.g., deep, technical divers) conservative diving practice, closing a PFO might, however, reduce the risk for DCS by a factor three.

## Conclusion

Based on this prospective analysis of divers blinded to their “RLS-status,” we suggest a Relative Risk of DCS for recreational diving with a RLS to be 1.42 (all DCS) to 3.02 (confirmed DCS). However, the absolute risk for confirmed DCS is low (2.53/10,000 dives all divers, 1.80/10,000 RLS negative and 5.46/10,000 RLS positive) and even more, most of these DCS cases happen outside what we would consider “safe recreational diving.”

We therefore confirm the need for comprehensive evaluation and expert counseling of each diver presenting with DCS and PFO and seeking advice whether this PFO should be closed (Germonpre, 2015; Smart et al., 2015; Wilmshurst, 2019). Any recommendation on “conservative diving” should be given according to internationally published “low-bubble” guidelines, not simply relying on the dive computer to make “no-decompression dives.” Systematic screening of recreational divers for RLS or PFO, in the absence of a history of DCS, remains in our opinion not warranted.

## Data Availability Statement

The original contributions presented in the study are included in the article/Supplementary Material, further inquiries can be directed to the corresponding author.

## Ethics Statement

The studies involving human participants were reviewed and approved by ethical committee approval was obtained (Bioethics Committee of the Belgian Defense Force Medical Staff, 2003), and divers signed an informed consent form prior to the testing. Each testing was performed individually, subjects going through the informed consent process one at a time. The patients/participants provided their written informed consent to participate in this study.

## Author Contributions

All authors listed have made a substantial, direct and intellectual contribution to the work, and approved it for publication.

## Conflict of Interest

The authors declare that the research was conducted in the absence of any commercial or financial relationships that could be construed as a potential conflict of interest.

## Publisher’s Note

All claims expressed in this article are solely those of the authors and do not necessarily represent those of their affiliated organizations, or those of the publisher, the editors and the reviewers. Any product that may be evaluated in this article, or claim that may be made by its manufacturer, is not guaranteed or endorsed by the publisher.
