# Supplementary material for: Increased Risk of Decompression Sickness When Diving With a Right-to-Left Shunt: Results of a Prospective Single-Blinded Observational Study (The “Carotid Doppler” Study)
Source: Front Physiol. 2021 Oct 29;12:763408. doi: 10.3389/fphys.2021.763408 (PMC8586212; doi:10.3389/fphys.2021.763408)
Supplement: Supplementary file 1 [file Presentation_1.pdf]

Dear DAN Europe Research Diver,

You have agreed to participate in the DAN Europe **Prospective study on the Risk of Right-to-Left Shunts (RLS) for Decompression Sickness (DCS) in Recreational Sports Divers** (also called the DSL "Carotid Artery Doppler Study").

You have undergone a Carotis Doppler Test approximately 5 years ago; now it's time to collect the final data in order to conclude your participation. This questionnaire is all it takes. It may require some effort to gather the data from your dive logs.

Please try to answer these questions as truthfully as possible. The answers will permit us to have a correct idea of your diving activity and any events that have happened to you during this period.
It will help us determine the statistical risk that you have been exposed to.

Each question asking for numbers will be followed by a checkbox called "exactly". Check this if you are 99% sure of the correct number (e.g. because you were able to calculate it from the dives log information). If you do not have the exact information but are estimating, leave the check-box "unchecked".

1. **Your identity:**

   a. Your DAN Research ID # (as on the card):
   b. Your name and birthdate:
   c. The date you have been tested (as on the card):
   d. Do you (still) smoke ? ☐ No ☐ Yes, cigarettes / day

2. **Your diving activity since your Carotis Doppler Test:**

   a. Total number of dives performed <u>since your Carotis Doppler Test</u>: ☐ Exactly
      Total dive time (hours and minutes) performed <u>since your test</u>: ...... ☐ Exactly
   b. Do you use a computer for diving ? : ☐ Yes ☐ No, I use tables
   c. Which computer or tables did you use mainly (> 75% of the time) ?
      Please write down brand and model of your computer (e.g.: Suunto Viper, Mares M1, Uwatec Aladin Air), or tables (e.g.: USNavy, DCIEM)

d. How many (proportion - %) of these dives were
("Decompression dive" is a dive where the computer, at one time during the dive, indicated that a decompression stop is obligatory. Even if this stop "disappears" later during the dive ! This does not include the "systematic safety stop" imposed by most modern computers):

i. "decompression air dives" : .... % ☐ Exactly
ii. "no decompression air dives": .... % ☐ Exactly
iii. "decompression Nitrox dives": .... % ☐ Exactly
iv. "no decompression Nitrox dives": .... % ☐ Exactly
v. were Nitrox dives using "Air Tables" or "Computer set on Air": .... % ☐ Exactly
vi. were done with other breathing gases or were technical dives (Trimix / Rebreather…): .... % ☐ Exactly
Please specify:

PLEASE NOTE : Total percentage of dives should to be 100% !

e. How many (proportion - %) of these dives were :

i. "recreational dives" (warm water, good visibility, long duration but not so very deep, usually < 30m)
(Typically: vacation/tropical dives): .... % ☐ Exactly
ii. "sports dives" (cold water, current, low visibility, usually deeper (25-35 m) but usually shorter)
(Typically: Zeeland or North Sea dives, even in summer): .... % ☐ Exactly
iii. "deep dives" (deeper than 35m, usually deco-dives using air as breathing gas or technical dives): .... % ☐ Exactly
iv. "low-risk dives" (no-deco dives <15m using air or <25msw using Nitrox, usually one dive per day) : .... % ☐ Exactly
v. Other:

PLEASE NOTE : Total percentage of dives should be 100% !

3. Possible or confirmed decompression sickness (DCS) during this period:

a. Have you been treated in a recompression chamber for decompression sickness during this period ? .... ☐ Yes ☐ No

b. **If NO, skip to question 4.**

c. If YES, describe your symptoms BEFORE treatment:
   i. ☐ Paralysis or weakness in one or two limbs
      1. ☐ Left leg
      2. ☐ Right leg
      3. ☐ Left arm
      4. ☐ Right arm
      5. ☐ Other :
   ii. ☐ Sensory disturbances (numbness, prickling)
      1. ☐ Left leg
      2. ☐ Right leg
      3. ☐ Left arm
      4. ☐ Right arm
      5. ☐ Body
      6. ☐ Face or neck
      7. ☐ Other :
   iii. ☐ Dizziness or hearing problem
   iv. ☐ Urinary difficulty or problems with passing stools
   v. ☐ Skin rash or "marbled skin", or itching or painful skin
      1. ☐ Left leg
      2. ☐ Right leg
      3. ☐ Left arm
      4. ☐ Right arm
      5. ☐ Body
      6. ☐ Face or neck
      7. ☐ Other :

d. Date, depth and duration of the dive that provoked the DCS:
   i. (date), m for minutes .... ☐ Exactly
   ii. How would you classify the "accident dive":
      1. ☐ "low-risk dive" (no-deco dive <15m using air or <25m using Nitrox, one dive per day)

2. ☐ "recreational dive" (warm water, good visibility, long duration, depth <30m)
3. ☐ "sports dive" (cold water, current, low visibility, 25-35 m)
4. ☐ "deep dive" (> 35m, deco-dive using air)
5. ☐ "technical dive" (using other breathing gases, cave dive or using rebreather)

iii. Were any "mistakes" made during the dive that could possibly explain your DCS ?

1. ☐ Missed deco-stops
2. ☐ Rapid ascent from depth
3. ☐ Diving while being ill or lack of sleep
4. ☐ Diving while on medication (please specify):

iv. How long after surfacing did the symptoms appear ?

1. ☐ Immediately
2. ☐ Between 5 minutes and 60 minutes after surfacing
3. ☐ Between 1 hour and 6 hours after surfacing
4. ☐ More than 6 hours after surfacing
5. ☐ After flying or high altitude

e. How many treatments in the recompression chamber did you receive before having no more symptoms at all ?

i. ☐ Only one
ii. ☐ Between 2 and 5
iii. ☐ Between 5 and 20
iv. ☐ More than 20

4. You have never been treated for DCS, but:
did you ever feel one of the following symptoms after a dive (which disappeared without recompression treatment) ?

a. ☐ Skin rash or marbled skin, or itching or painful skin
b. ☐ Dizziness or hearing problem
c. ☐ General fatigue, unusual for the dive that you just did (exhaustion)
d. ☐ Numbness, pain or difficulties moving one of your limbs

e. If you checked one of the above, please describe below the symptoms as good as you can:

f. Please note here the details of the dive that provoked these symptoms:

   i. (date), m for minutes .... ☐ Exactly

   ii. ☐ Decompression dive

   iii. ☐ Repetitive dive (less than 6 hours after previous dive)

   iv. ☐ Missed deco-stops

   v. ☐ Rapid ascent

   vi. ☐ Diving while being ill or lack of sleep

   vii. ☐ Diving while on medication (please specify):

g. What treatment did you do and how long did it take for the symptoms to disappear completely ?

   i. Treatment (describe as good as possible) :

   ii. Symptoms disappeared after :

      1. ☐ Some minutes
      2. ☐ Some hours
      3. ☐ Some days
      4. ☐ Did not disappear, are still present

Thank you for filling in this questionnaire !
If you have any more remarks or questions, please write them here:

Please send this questionnaire by e-mail to pgermonpre@daneurope.org or by mail to

Dr Peter Germonpre
Centre for Hyperbaric Oxygen Therapy
Military Hospital Brussels
Rue Bruyn 1
1120 Brussels – Belgium
Or by Fax to +32 2 264 4861 (attn. Dr P. Germonpré)

Now, you can choose to stop or continue the study. If you want to be informed of the result of the Carotis Doppler Test that you did 5 years ago, we will send you the results by mail or e-mail.

☐ No, do not inform me of the result, I would like to continue the Carotis Doppler Study for another 5 years and help you collect more dives. I will receive a new questionnaire then.

☐ Yes, I would like to know the result of my Carotis Doppler Test (and stop the participation in the study) . Please send it to the following address or e-mail:

☐ No, please do not send me the result. I want to stop the study now, but I do not wish to know the result.

Thank you again for your precious collaboration !
