# Supplementary material for: Increased Risk of Decompression Sickness When Diving With a Right-to-Left Shunt: Results of a Prospective Single-Blinded Observational Study (The “Carotid Doppler” Study)
Source: Front Physiol. 2021 Oct 29;12:763408. doi: 10.3389/fphys.2021.763408 (PMC8586212; doi:10.3389/fphys.2021.763408)
Supplement: Supplementary file 2 [file Data_Sheet_1.pdf]

Supplemental material :

1. Final Questionnaire

   See : Final Questionnaire

2. Cases description

   a. RLS-positive cases
      i. HBO treated (4/8)
         1. 58msw 29minutes on air; amaurosis left eye + LL/pelvis weakness
         2. 54msw 30minutes on air; deafness left ear
         3. 36msw 50minutes bottom on CCR; oedema left arm + cutis marmorata
         4. 65msw 50minutes bottom time on air: vertigo + visual problems + skin rash 5 minutes after surfacing
      ii. Not HBO treated (4/8) + 1 case possible DCS
         1. 99msw on air; severe vertigo/nausea + amaurosis one eye, “difficulty thinking” – was diving alone, went home, all went better after about 20 hours
         2. 30msw 45minutes on air – repetitive decompression dive; hemi-anopsia + blurred vision, skin rash (*NB multiple instances of skin rash after diving*)
         3. 62msw 68minutes on CCR; skin rash
         4. 39msw 31minutes on air – square decompression dive cold water; weakness legs + urinary problems
         5. 20msw for 45 minutes on air, some minutes of dizziness after surfacing, no treatment except rehydration
   b. RLS-negative cases
      i. HBO treated (6/10)
         1. 4 were technical dives (deep Trimix OC) – symptoms were rash (3/4), motor (2/4), sens (1/4), vertigo (1/4)
         2. 2 were cold water square decompression cold water dives on air – symptoms were rash (1/2), vertigo (2/2), visual (1/2)
      ii. Not HBO treated (4/10) + 9 cases possible DCS
         1. 5 had skin rash only, appearing 5-60minutes after dive
         2. Average dive depth 40.76msw (25-110)
         3. 6 were decompression or repetitive dives
         4. Only 2 (skin rash) applied oxygen first aid
